# Loss of *CDX2* and high COX2 (*PTGS2*) expression in metastatic colorectal cancer

**DOI:** 10.3332/ecancer.2024.1666

**Published:** 2024-02-08

**Authors:** Álvaro M C Caldas, Warley A Nunes, Rodrigo Taboada, Marcelle G Cesca, Janaína N Germano, Rachel P Riechelmann

**Affiliations:** 1 Department of Clinical Oncology, AC Camargo Cancer Center, São Paulo 01509-900, Brazil; 2 Department of Pathology, AC Camargo Cancer Center, São Paulo 01509-900, Brazil; 3 Statistic Group at the International Research Center (CIPE), AC Camargo Cancer Center, São Paulo 01509-900, Brazil

**Keywords:** CDX2, COX2, BRAF, metastatic colorectal cancer, chemotherapy

## Abstract

Lack of expression of the tumour suppressor gene caudal-type homeobox 2 (*CDX2*) associates with poor outcomes in early stage colorectal cancer (CRC). Yet its prognostic value in the context of other prognostic biomarkers in metastatic CRC (mCRC) is unknown. Overexpressed cyclooxygenase-2 (COX2) has been reported in advanced CRC. However, CDX2 and COX2 relationship in mCRC remains undetermined. We aimed to assess their expression in mCRC tumours from a clinically characterised cohort and their influence on overall survival (OS) and progression-free survival (PFS) in first line. Among 720 consecutive mCRC patients, 346 had tumour samples appropriate for tissue microarray assembly and immunohistochemistry analyses. Clinical and survival data were retrospectively assessed. Loss of *CDX2* expression was detected in 27 (7.8%) samples, enriched in poorly differentiated tumours (20%; *p* < 0.01) and in those with the *BRAF* p.V600E variant (40%; *p* < 0.01). Most tumours (93.4%) expressed COX2. COX2-negative samples were enriched in poorly differentiated mCRC. In unadjusted analyses, median OS (*p* < 0.001) and median PFS (*p* < 0.05) were inferior for patients with CDX2-negative versus CDX2-positive tumours. In conclusion, loss of *CDX2* was significantly associated with poorly differentiated mCRC and *BRAF *p.*V600E* allele and a prognostic marker of worse OS.

## Introduction

Significant advances have been achieved towards personalised treatment for patients with metastatic colorectal cancer (mCRC) [37]. While specific gene alterations are important to guide targeted therapy, they are not sufficient to determine a patient’s clinical outcome. For instance, while *RAS* mutations in mCRC are negative predictors of response to anti-epidermal growth factor receptor (EGFR) monoclonal antibodies (mAb) [9, 49], we still lack more accurate predictors of benefit to anti-EGFR agents.

The *BRAF* p.V600E variant, which hyperactivates the EGFR pathway, is a strong negative prognostic factor in mCRC [40]. Nevertheless, combined *BRAF* and *KRAS* inhibition is required in order to control adaptive resistance due to feedback loops mediated by colorectal cancer (CRC)-specific EGFR activity [11]. The identification of deficient mismatch repair in mCRC, ascertained by laboratorial identification of DNA microsatellite instability (MSI) in tumours, selects patients who may benefit from immune checkpoint inhibitors. Thus, nowadays testing for mismatch repair (MMR) status, *KRAS*, *NRAS* exons 2, 3 and 4, and *BRAF* p.V600E gene alterations has been recommended at the time of mCRC diagnosis, due to its relevance for first-line therapy selection [6]. More recently genomic amplification of the human epidermal growth factor receptor 2 (*HER2*) gene [41] has been recommended for wild-type *RAS* tumours, contributing to identify patients who may benefit from HER2 blockade [6]. Although *NTRK* fusions are rare in mCRC, tropomyosin receptor kinase inhibitors are also recommended as a targeted therapy [6]. Notwithstanding, despite all achievements, most patients with mCRC are still incurable. Thus, investigation of new biomarkers is needed to refine prognosis and response prediction, notably with routine methods and appealing low cost.

There are currently several emerging prognosis and/or predictive biomarkers under evaluation in mCRC [35]. Among them, the caudal-type homeobox transcription factor 2 (CDX2), a protein encoded by the *CDX2* gene (13q12.2; OMIM #600297), plays an essential role in the development and differentiation of intestinal epithelium, regulating the expression of intestine-specific genes, many of them associated with cell proliferation, migration and tumorigenesis [7, 13, 16, 19, 27, 44]. Loss of *CDX2* function associates with oncogenesis, a property of tumour suppressor genes [8]. A variable rate of low CDX2 amount has been observed in non- mCRC. It appears to correlate with inferior survival, tumour poor differentiation, proximal location, MSI, CpG island methylator phenotype and *BRAF* pathogenic variants, notably the p.V600E variant [3, 4, 10, 26, 48, 50, 57]. However, the independent prognostic value of CDX2 downregulation in CRC is still under debate. Few reports have solely addressed CDX2 expression and its potential prognostic and predictive values in mCRC [1, 54, 55]. Thus, further investigation in different patient cohorts is necessary to validate these findings and to investigate the prognostic association of other factors with CDX2.

Chronic inflammation is a known risk factor for the development and outcome of CRC [18]. CDX2 loss has been linked to intestinal inflammation as an upstream regulator of key molecules in the inflammatory signalling cascade. Reduced CDX2 in a human colon cancer cell line has been demonstrated to enhance NF-κB-mediated inflammatory response, upregulating the expression of cyclooxygenase 2 (COX2) protein, encoded by the prostaglandin (PG)-endoperoxide synthase 2 (*PTGS2*) gene (1q31.1; OMIM #600262). In this case, COX2 overexpression is due to reduced binding of CDX2 to NF-κB. This protein-protein association is sufficient to impair NF-κB transactivation of the PTGS2 promoter [29]. Conversely, NF-κB has an opposite effect on the CDX2 promoter if associated with the pro-inflammatory cytokine, tumour necrosis factor-alpha, repressing its activity thus decreasing the CDX2 protein amount [22]. Hence, on the one hand, inflammation can be down-regulated by the CDX2 protein. On the other hand, the CDX2 promoter can be repressed by certain cytokines, reducing this gene’s transcription [8]. COX2 catalyses the conversion of free arachidonic acid to PG H2, the precursor of other PGs and thromboxanes. These compounds are important regulators of cell proliferation, angiogenesis, immune function and inflammation, which may contribute to the development and progression of neoplasia [15]. Although COX2 overexpression has shown a significantly direct association with CRC recurrence [23, 51] and advanced CRC stages [56], its overall prognostic significance in CRC remains unclear [36].

Here, we aimed at better understanding the role of CDX2 and COX2 biomarkers on the clinical outcomes of patients with mCRC and their associations with other known prognostic factors in CRC. For that, we retrospectively assessed a clinically characterised cohort of mCRC patients from a large comprehensive cancer centre, and chronologically classified the expression of CDX2 and COX2 in tissue microarray (TMA) of mCRC tumour samples.

## Subjects and methods

### Patient selection and study design

This was a retrospective longitudinal study, which aimed to evaluate the immunohistochemistry (IHC) expression of both CDX2 and COX2 proteins in colorectal tumour samples and their effect on patients’ overall survival (OS). The study was conducted in accordance with the ‘International Conference on Harmonization Good Clinical Practice’ protocol guidelines as well as applicable local laws and regulatory requirements. The study was reviewed and approved by the Research Ethics Committee of the AC Camargo Cancer Center, São Paulo, Brazil (CAAE 06432819.2.0000.5432; http://plataformabrasil.saude.gov.br). This retrospective, single-centre cohort study had data available for 720 patients with mCRC. Inclusion criteria were: adult patients treated at AC Camargo Cancer Center (São Paulo, Brazil) with metastatic disease diagnosed between January 2015 and December 2019, and with available tumour tissues from primary tumour and/or metastatic lesions suitable for analysis according to information provided by the Department of Pathology. Exclusion criteria were patients with insufficient tumour tissues for CDX2/COX2 analysis, and scant available clinical history (e.g., patients consulted for second opinion).

The primary endpoint was OS of mCRC patients according to the tumour expression status of CDX2 or COX2. Secondary endpoints were: progression-free survival (PFS), frequency of CDX2 and COX2 protein expression by IHC in mCRC (positive/negative); to investigate associations between CDX2 and COX2 protein expression and tumour-related factors such as, presence of *KRAS*, *BRAF* or *NRAS* mutations (*KRAS* exons 2, 3 and 4 (codons 12, 13, 59, 61, 117 and 146), *BRAF* exon 15 (codon 600), and *NRAS* exons 2, 3 and 4 (codons 12, 13, 59, 61 and 117)); proficiency of proteins related to repair genes (*MLH1*, *MSH2*, *MSH6*, *PMS2*); tumour sidedness and cell differentiation.

Clinical data collected from patients' electronic medical records were entered and stored in a data collection form developed within the Research Electronic Data Capture software (REDCap, Vanderbilt University, Nashville TN; REDCap data bank 2664/19 at CIPE/A.C. Camargo Cancer Center). The following data were collected and analysed: age at the diagnosis of mCRC, gender, family history of colorectal neoplasia, known hereditary syndrome, co-morbidities (only those requiring pharmacological treatment), tumour location (right colon defined as localised tumour from ascending colon to splenic flexure), morphology, grade, date of mCRC diagnosis, location of metastases, genotypes for *MLH1*, *MSH2*, *MSH6*, *PMS2*, *KRAS*, *BRAF* and *NRAS* genes; classification of protein proficiencies related to DNA repair genes *MLH1*, *MSH2*, *MSH6*, *PMS2*; type and date of initiation of first-line chemotherapy regimen with or without a biologic agent, date of metastatic disease progression after first-line chemotherapy (with or without a biologic agent), number of chemotherapy cycles, date of last follow-up visit, and date of death.

### TMA, CDX2 and COX2 IHC

Haematoxylin and eosin-stained representative tumour samples were reviewed by a senior pathologist who selected a subset for microarray assembly. Paraﬃn-embedded tissue blocks retrieved from the primary tumour (79.8%) or metastatic lesion (20.2%) had to provide two 1-mm diameter spots for capture. For each tumour, two independent tissue cores from distinct areas of the same lesion were sectioned. Each spot represented viable tissue of invasive neoplasia for construction of a TMA for each marker employed in this study. TMA assembly was performed according to standards used in the Human Protein Atlas [21].

IHC was performed using the anti-CDX2 (EPR2764Y) and anti-COX2 (SP21) antibodies from Abcam (Cambridge, UK), on an automated platform (Ventana Benchmark, Roche Diagnostics, Rotkreuz, Switzerland), following the manufacturer's instructions. A qualified normal human term placenta tissue was used as internal control for each slide.

Stained tissue was morphologically evaluated at forty- and a hundred-fold magnification. Subjective scoring of CDX2 signal intensity was performed at two-hundred magnification in a blinded fashion [10]. Immunoreactivity was assigned as a score based on the percentage of positive tumour cells over total tumour cells (proportion of positivity) ranging from 0% to 100%. The recommendation for general IHC interpretation of CRC markers is to consider the percentage of positive cells as the only scoring parameter [58]. Thus, positivity, but not intensity, of CDX2 IHC staining was taken into account. Loss of CDX2 expression (CDX2-negative) was deﬁned as tumours with the malignant epithelial component completely lacking CDX2 expression or showing a scattered, faint nuclear staining in a minority fraction of cancer cells (weak staining). CDX2-positive samples were all tumours with malignant epithelial component displaying widespread nuclear expression of CDX2 (moderate staining) or having a strong staining in a majority of cancer cells (strong staining). When CDX2 scores were discordant between the two TMA tumour cores, the highest score was considered.

After all slide sections were scanned by the pathologist, a score was assigned to COX2 staining, which considered both staining intensity and extent of stained area. The staining intensity was scored as 0 (negative), 1 (weak), 2 (medium), or 3 (strong). The extent of staining was scored as 0 (0%), 1 (1% to 25%), 2 (26% to 50%), 3 (51% to 75%) or 4 (76% to 100%), according to the proportion between stained areas and the whole tumour area, as proposed [47]. The sum of the intensity and extent score was used as the final staining score (0 to 7). For statistical analysis, tumours having a final staining score ≥3 were considered COX2-positive [47].

### Statistics considerations

Initially, a descriptive analysis of the variables was performed, in which the absolute (*n*) and relative (%) frequency distributions were presented for the qualitative variables, and the main summary measures, such as the mean, SD, median, minimum and maximum values, for quantitative variables. To assess the association between qualitative variables, the chi-square test or Fisher's exact test were used when appropriate. To compare qualitative variables in relation to the distribution of quantitative variables, the non-parametric Mann-Whitney test was used. OS was calculated from the date of C1D1 of first-line treatment until the date of death from any cause. PFS was considered the time from the date of C1D1 treatment to radiological progression, or death from any cause. The Kaplan-Meier estimator was used to estimate the OS and PFS curves, and the groups were compared using the log-rank test.

Univariate and multivariate Cox proportional hazards models were created to adjust the effects of independent variables on the primary endpoint of OS. The independent variables used were CDX2 expression (positive vs. negative), *BRAF* (p.V600E variant versus wild-type allele) and tumour sidedness (right versus left/rectum). Variables from the univariate model were incorporated into the multivariate Cox proportional hazard model if *p* < 0.20. Statistical analysis was performed by SPSS software (Version 28, IBM, Armonk NY) and free software R (Version 4.1.3, R Foundation, Vienna, Austria). Two-tailed *p* < 0.05 was considered signiﬁcant.

## Results

### Study subjects

Among 720 mCRC patients selected for initial assessment, 346 had tumour tissues appropriate for TMA assembly. The remaining had either no viable tumour tissues (*N* = 291; i.e., scant biopsy material) or upon stained scoring evaluation there was no viable stained image or tumour was missing (*N* = 83). Therefore, this study analysed demographic data and tissue samples of 346 mCRC patients that had been treated at our institution from 2015 to 2019. The characteristics of patients are summarised in Table 1.

**Figure 1.**
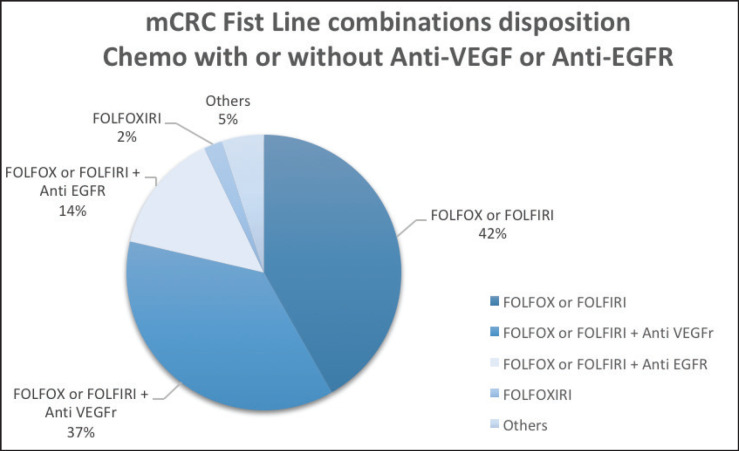
Plot of the frequency of combined first-line chemotherapy of 331 mCRC patients.

The median OS for all patients (*N* = 346) was 51 months (31.3–70.7). The median PFS in first-line chemotherapy (with or without a mAb) was 11 months (9.7–12.3). Information on ﬁrst-line treatment was available for 331 patients (Figure 1; Table 1).

### CDX2 and COX2 expression in mCRC

**Figure 2.**
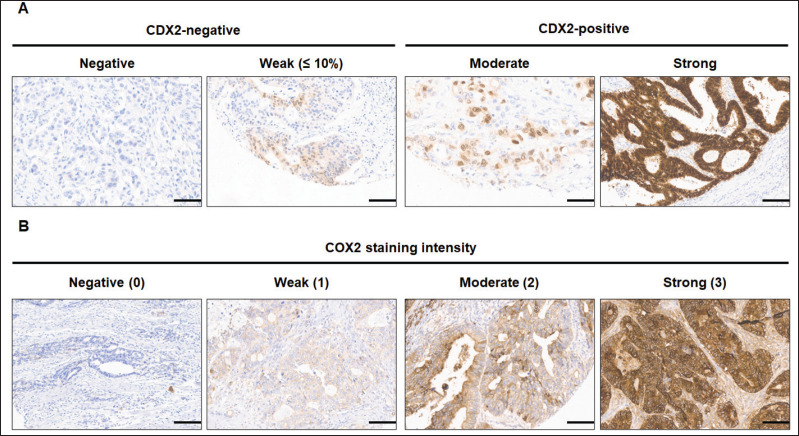
Immunohistochemical staining images of CDX2 and COX2 of mCRC tumour TMA. (A) CDX2 staining was deﬁned as CDX2-negative tumours in which the malignant epithelial component completely lack CDX2 expression (negative) or show a scattered, faint nuclear staining (weak, ≤10%) in a minority fraction of cancer cells. (B) COX2 staining intensity was negative (score 0), weak (1), moderate or strong (3). Scale bar: 35 μm. Original magnification ×200.

All 346 patients had CDX2 expression status ascertained on tissue. The classification of CDX2 staining (Figure 2) disclosed 27 patients (7.8%) whose tumours lacked CDX2 expression and 319 (92.2%) with CDX2-positive tumours. There were no significant differences for age or gender according to CDX2 tumour expression (Table 2).

There was a significant association between grade-3 tumours and loss of CDX2 expression (Table 2). All CRC samples in our cohort had been genotyped for the *KRAS* or *NRAS* genes. CDX2-positive or CDX2-negative status were equally represented in tumours with wild-type (*N* = 178, 51.4%) or mutated (*N* = 168, 48.6%) *KRAS* or *NRAS*. The *BRAF* p.V600E variant was more frequent among CDX2-negative tumours, detected in six (40%) of them as compared to nine (60%) CDX2-positive samples (Table 2). No significant association was found between the CDX2 expression status and MMR deficiency or metastatic sites (Table 2).

**Figure 3.**
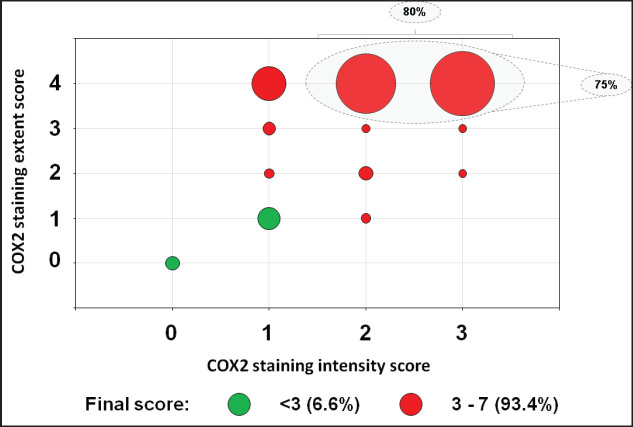
Bubble plot distribution of the COX2 staining in 332 mCRC samples. COX2 final score was the sum of COX2 staining intensity and extent scores. The staining intensity score was defined as 0 (negative), 1 (weak), 2 (moderate) or 3 (strong). The staining extent score was classified as 0 (0%), 1 (1%–25%), 2 (26%–50%), 3 (51%–75%) or 4 (76%–100%).

COX2 expression was evaluated in 332 out of 346 tumour samples (Figure 2). COX2-positive tumours encompassed the majority (93.4%) of samples (Table 3), and the staining was extensive in most of them (86.4%, Figure 3). COX2-negative tumours were enriched in poorly differentiated tumours when compared to COX2-positive tumours (Table 3). All tumours that lacked CDX2 expression or had the *BRAF* p.V600E variant were positive for COX2 expression (Table 3).

### OS and PFS of mCRC patients

**Figure 4.**
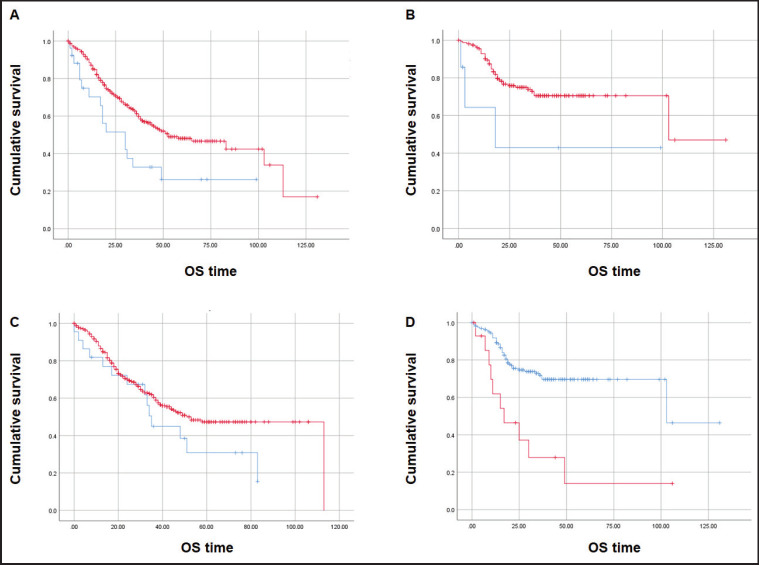
Kaplan-Meier estimates for OS curves. (A) Inferior OS for CDX2-negative (*N* = 27, blue) and CDX2-positive (*N* = 319, red) patients. (B) Lower OS for CDX2-negative (*N* = 7, blue) compared to CDX2-positive (*N* = 157, red) patients without the *BRAF* p.V600E variant. (C) Similar OS between COX2-negative (*N* = 22, blue) and COX2–positive (*N* = 309, red). (D) Significant OS difference for mCRC patients – *BRAF* p.V600E variant (*N* = 15, red) and wild-type allele (*N* = 164, blue). Mantel-Cox log-rank test significant (*p* < 0.05) for (A), (B) and (D).

At a median follow-up time of 51 months, the median OS was inferior for patients with CDX2-negative tumours (*N* = 27) when compared with those with CDX2-positive CRC (*N* = 319): median 30 versus 53 months, respectively (Mantel-Cox log-rank test, *p* = 0.008; Figure 4A). Inferior OS was also observed for patients with CDX2-negative (*N* = 8) compared to CDX2-positive (*N* = 157) tumours when adjusted to exclude samples with the *BRAF* p.V600E variant (median 18 versus 103 months, respectively; Mantel-Cox log-rank test, *p* = 0.043; Figure 4B). No OS difference was observed between COX2-negative (*N* = 22) and –positive (*N* = 309) sample patients (*p* = 0.14; Figure 4C). Irrespectively of CDX2 or COX2 expressions, there was a significant OS difference between mCRC patients with *BRAF* p.V600E variant (*N* = 15) and those with the wild-type allele (*N* = 164) (median 17 and 103 months, respectively, Mantel-Cox log-rank test, *p* = 0.0001; Figure 4D). Multiple Cox regression analysis performed to adjust for prognostic covariates (*BRAF* genotype and tumour sidedness) disclosed a significant decrease in OS for patients with the p.V600E variant (*p* = 0.029) but not with loss of CDX2 expression (Table 4).

**Figure 5.**
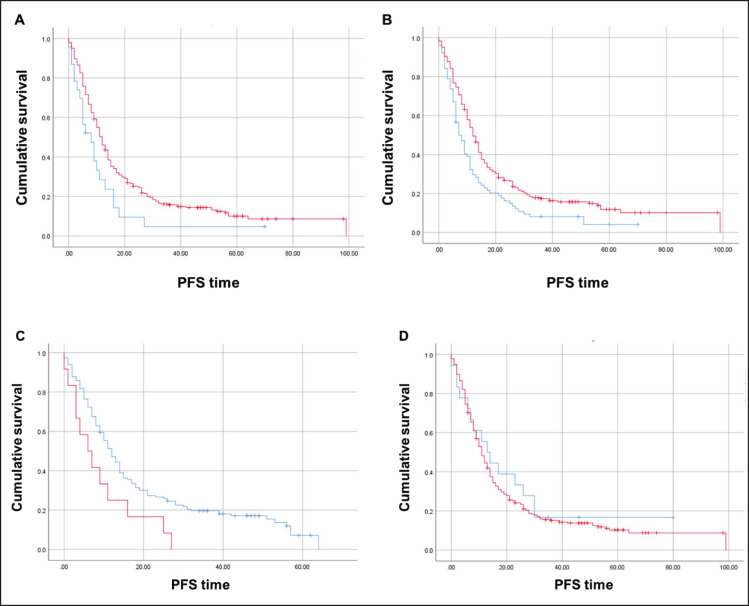
Kaplan-Meier estimates for PFS curves. (A) Significant difference for CDX2-negative (*N* = 23, blue) compared to CDX2-positive (*N* = 282, red) tumours (Mantel-Cox log-rank test significant (*p* < 0.05). (B) Significant difference for right sided (*N* = 76, red) compared to left/rectal (*N* = 228, blue) tumours (Mantel-Cox log-rank test significant (*p* < 0.002). (C) Significant difference for mCRC patients – BRAF p.V600E variant (*N* = 12, red) compared to wild-type allele (*N* = 148, blue) (Mantel-Cox log-rank test significant (*p* < 0.021). (D) No difference between COX2-negative (blue) and COX2-positive (red) patients.

The median PFS in first-line therapy was significantly inferior for those with CDX2-negative (*N* = 23) compared to CDX2-positive (*N* = 282) tumours (8 versus 12 months; Mantel-Cox log-rank test, *p* = 0.023; Figure 5A). When we stratified by tumour sidedness, PFS in first-line was significantly *i* for those located on the right side (*N* = 76 ) compared to left side/rectal (*N* = 228) tumours (7 versus 12 months; Mantel-Cox log-rank test, *p* = 0.002; Figure 5B). A significant difference was also found for mCRC patients with *BRAF* p.V600E variant (*N* = 12, red) compared to those wild-type allele tumours (*N* = 148, blue), (6 versus 12 months; Mantel-Cox log-rank test, *p* = 0.021, Figure 5C). COX2 expression status revealed no effect in PFS (*p* > 0.05; Figure 5D).

## Discussion

In a cohort of clinically characterised patients with mCRC 7.8% of samples lacked CDX2 expression. CDX2-negative samples were significantly associated with the *BRAF* p.V600E variant and poorly differentiated histology. Median OS and PFS were significantly inferior for patients with CDX2-negative versus CDX2-positive tumours, even after excluding tumours with the *BRAF* p.*V600E* allele. CDX2 expression was not associated with tumour sidedness. Tumours overly expressed COX2 but it had no influence on patients’ OS.

The CDX2 expression status has a variable prevalence in mCRC. In unselected mCRC, the frequency of CDX2 loss varies from 2.8% to 48% [3, 31]. This variability may be explained, among other reasons, by differences in the threshold of CDX2-negative samples. While some studies classified as negative the complete absence of CDX2 IHC expression [55], other studies considered samples as negative when there was weak nuclear expression in less than 10% tumour cells [10]. One report employed a score combining the intensity level and the percentage of tumour cells expressing CDX2, categorizing samples with low, intermediate and high expression levels [32]. In our study, CDX2-negative samples had none or weak nuclear staining in up to 10% of tumour cells, whereas CDX2-positive tumours had moderate or strong staining (Figure 2).

In our cohort the histopathological classification of grade 3 was more frequent in CDX2-negative tumours (20%) than expected (7.8%; *p* < 0.01), as observed by previous studies and linked to a poor prognosis in mCRC [1, 3, 4, 14, 45, 55]. However, since grade-3 tumours comprised 9.4% (*N* = 30) of the total sample, grade-3 CDX2-negative tumours corresponded to six samples of our cohort. This significant enrichment though with a limited number of samples was also observed in other studies [31, 43].

We found a significant enrichment in the *BRAF* p.V600E variant in CDX2-negative samples when compared to CDX2-positive samples (40% versus 60%, respectively; Table 2). A significant association has been reported between CDX2 loss and *BRAF* p.V600E [1, 3, 4, 31] or MSI [1, 31]. Some studies observed that *KRAS* mutations more frequently in tumours with CDX2 expression [1], while other studies did not [55].

*BRAF* p.V600E and loss of CDX2 might cooperate in promoting CRC tumorigenesis. In a cohort of mCRC* BRAF V600E* samples, 67% had low to intermediate intensity of CDX2 staining [25]. Mouse models with *Cdx2* null and *Braf* p.V600E alleles develop intestinal tumours with serrated phenotype and synergistic gene expression effects, such as a hundred-fold increase in the amount of the gastric epithelium marker Anx10a protein [38].

The rate of CDX2 loss was significantly increased in right-sided primary tumours when compared to CDX2-expressing tumours from two studies [1, 55]. However, in our study, we could not observe this association, potentially due to our smaller sample.

### CDX2 and mCRC clinical outcomes

In early stage CRC, the lack of CDX2 expression has been associated with poor patient prognosis and limited OS in different studies [10, 17, 50]. In a subgroup of patients with high-risk stage-II CRC, loss of CDX2 was proposed as a predictive biomarker for treatment benefit from adjuvant chemotherapy [10]. In our casuistic, loss of CDX2 expression in mCRC was associated with a higher risk of death, even when wild-type *BRAF* samples were separately assessed. Patients with CDX2-negative tumours additionally had a faster progression on first-line treatment than those with tumours that expressed CDX2. Lack of CDX2 was a poor prognostic factor for mCRC in other retrospective studies [1, 5, 46, 54, 55].

Importantly, the prognostic effect of CDX2 has been investigated in the presence of other known prognostic biomarkers. *BRAF* p.V600E variant confers significantly worse prognosis for mCRC independently of associated clinic-pathological features [42, 52]. Although there are discrepancies among different series, CDX2 down-regulation is commonly correlated with the *BRAF* p.V600E variant [1, 4, 34], as we observed. When multiple Cox regression analysis was employed to adjust for covariates, reduced OS was observed for patients with the *BRAF* p.V600E variant but not with CDX2 loss (Table 4), demonstrating that in our cohort the lack of CDX2 was not an independent outcome predictor or that our study lacked power to detect this association. Other studies identified the absence of expression of CDX2 as an independent poor prognostic marker in mCRC [1, 25, 39]. Similarly to other studies, we observed that loss of CDX2 expression was associated with a significantly higher risk of progression after first-line treatment [1, 54].

The present study is to our knowledge the largest IHC analysis of COX2 expression in mCRC, and the first one to assess its correlation with CDX2 protein levels. In cohorts with mixed stages of CRC, at least 60% of metastatic tumours highly expressed COX2 [2, 12, 33, 47]. The study on which we based our COX2 staining cutoff examined its expression in CRC, mCRC, and normal mucosa [47]. Similar to their CRC samples, we had nearly 80% of the samples with moderate (score 2) to strong (score 3) staining intensity (Figure 3). While in other series, COX2 expression was not significantly different among tumour grades [12, 47], a significant enrichment of poorly differentiated tumours was observed among COX2-negative samples in our study. Different studies reported higher OS rates among CRC patients whose tumours are negative for COX2 protein [24, 28, 30, 36, 47, 59]. In our study, the lack of significant difference between the two groups for either OS or PFS is probably compromised by the small number of COX2- negative samples.

The present study had some limitations, many of which inherent to its retrospective designs. We could not fully explore the relationship between CDX2 expression status and overall response rate and response to specific first-line therapies. This would be of particular interest for anti-EGFR agents.

Immunohistochemical detection of CDX2 expression is recognised as a clinically useful diagnostic biomarker in CRC specially in those metastatic cases where tumour origin is unknown [3, 20, 53]. Our results corroborate the clinical relevance of CDX2 status as a prognostic biomarker in mCRC and we think it should be routinely integrated into clinical practice. Furthermore, It should be certainly incorporated into prospective clinical trials alone or in combination to new and/or existing biomarkers exploring potential synergies and its value as a predictive biomarker also in the mCRC setting.

## Conclusion

The loss of CDX2 expression in mCRC was associated with a higher risk of death and progression after first-line treatment, and with poorly differentiated tumours, and the somatic BRAF p.V600E variant. Although, in the absence of the BRAF p.V600E variant, the lack of CDX2 protein was also associated with inferior OS, the loss of CDX2 did not emerge as an independent predictor. Our data preliminary supports the validity of this biomarker to distinguish cases that may potentially warrant a more aggressive therapeutic approach. Further investigation of CDX2 in future prospective clinical trials as a prognostic stratifying factor may help guide better therapeutic management for these patients.

## Conflicts of interest

The authors declare that they have no conflicts of interest to disclose regarding the publication of this manuscript.

## Funding

An independent grant from Amgen Brazil has enabled tumour tissues handling, lab equipment, and IHC analyses. AC Camargo Cancer Center, São Paulo, Brazil, provided resources for statistical analysis, data collection and database assembly, and staff support for regulatory processes.

## Clinical practice points

- Lack of expression of the tumour suppressor gene *CDX2* associates with poor outcomes in early-stage CRC, but few reports have solely addressed its potential prognostic and predictive values in mCRC. Chronic inflammation is a known risk factor for the development and outcome of CRC and overexpressed COX2 has been reported in advanced CRC.
- Loss of CDX2 expression in mCRC was associated with higher risk of patient death and progression after first-line treatment, poorly differentiated tumours and the *BRAF* p.V600E variant.
- In the absence of *BRAF* p.V600E variant, the lack of CDX2 protein was still associated with inferior OS.
- As COX2 was predominantly expressed in mCRC, no significant difference was detected in either OS or PFS.

## Figures and Tables

**Table 1.** Treatment history of patients with mCRC stratified by tumour and CDX2 expression.

Drug/Composites of first-line chemotherapy	Number of patients	Tumour samples	
		CDX2-negative	CDX2-positive
Total	331	27 (8.2%)	304 (91.8%)
FOLFOX/FOLFIRI	135 (40.9%)	7 (5.2%)	128 (94.8%)
Doublet + Bevacizumab	119 (35.9%)	8 (6.7%)	111 (93.3%)
Doublet + Anti-EGFR	47 (13.9%)	4 (8.7%)	43 (91.3%)
CAPOX (Capecitabine and Oxaliplatin)	8 (2.4%)	3 (37.5%)	5 (62.5%)
Triplet	7 (2.1%)	2 (28.6%)	5 (71.4%)
Other	16 (4.8%)	3 (18.7%)	13 (81.3)

Data presented as *n* (%)

FOLFIRI: folinic acid, 5-ﬂuorouracil, irinotecan; FOLFOX: folinic acid, 5-ﬂuorouracil, oxaliplatin; FOLFOXIRI: folinic acid, 5-fluorouracil, oxaliplatin and irinotecan; NA: not applicable

**Table 2.** Patient and tumour characteristics according to CDX2 expression (positive or negative) in mCRC.

Patient and tumour characteristics	Patients with analysed CDX2 expression	CDX2 expression status		CDX2 status comparison
		Negative CDX2	Positive CDX2	*p* value
Total number	346	27 (7.8%)	319 (92.2%)	
Median age at diagnosis of metastatic disease (95% CI)	59.7 (30–88)	58.7 (34–86)	59.7 (30–88)	1.00^a^
Male	185 (53.5%)	14 (8%)	171 (92%)	1.00^a^
Female	161 (46.5%)	13 (8%)	148 (92%)	
Histology	319	23 (7.2%)	296 (92.8%)	
Tumour grade 1 (Well differentiated)	25 (7.8%)	3 (12%)	22 (88%)	0.007^a^
Tumour grade 2 (Mod. differentiated)	264 (82.8%)	14 (5.3%)	250 (94.7%)	
Tumour grade 3 (Poorly differentiated)	30 (9.4%)	6 (20%)	24 (80%)	
Tumour sidedness	344	27 (7.8%)	317 (92.2%)	
Right sided	87 (25.3%)	10 (11.5%)	77 (88.5%)	0.187^a^
Left sided/Rectum	257 (74.7%)	17 (6.6%)	240 (93.4%)	
Tumour genetics				
*BRAF*	180	14 (7.8%)	166 (92.2%)	
Wild-type	165 (91.7%)	8 (4.8%)	157 (95.2%)	0.0002^a^
p.V600E variant	15 (8.3%)	6 (40%)	9 (60%)	
*KRAS* or *NRAS*	346	27 (7.8%)	319 (92.2%)	
Wild-type	178 (51.4%)	15 (8.4%)	163 (91.6%)	0.807^a^
Mutated	168 (48.6%)	12 (7.1%)	156 (92.9%)	
MMR	279	15 (5.4%)	264 (94.6%)	
MSS	270 (96.8%)	14 (5.2%)	256 (94.8%)	0.396^a^
High MSI	9 (3.2%)	1 (11.1%)	8 (88.9%)	
Sites of metastasis at diagnosis	346	27 (7.8%)	319 (92.2%)	
Liver	153 (44.2%)	12 (7.8%)	141 (92.2%)	0.287^a^
Peritoneum	74 (21.4%)	8 (10.8%)	66 (89.2%)	
Lung	59 (17.1%)	1 (1.7%)	58 (98.3%)	
Non regional lymph nodes	34 (9.8%)	3 (8.8%)	31 (91.2%)	
Bone	2 (0.6%)	0	2 (100%)	
Other	24 (6.9%)	3 (12.5%)	21 (87.5%)	

Data presented as *n* (%)

a Chi-square test

MMR: mismatch repair; MSS: microsatellite stability; MSI: microsatellite instability

**Table 3.** Patient and tumour characteristics according to COX2 expression (positive or negative in mCRC).

Patient and tumour characteristics	Patients with analysed COX2 expression	COX2 expression status		COX2 status comparison
		Negative COX2	Positive COX2	*p* value
Total number	332	22 (6.6%)	310 (93.4%)	
Median age at diagnosis of metastatic disease (95% CI)	59.5 (30–88)	58.6 (32–85)	59.5 (30–88)	
Male	177 (53.3%)	12 (6.8%)	165 (93.2%)	1.00^a^
Female	155 (46.7%)	10 (6.5%)	145 (93.5%)	
CDX2 expression status	332	22 (6.6%)	310 (93.4%)	
CDX2-negative	26 (7.8%)	0 (0.0%)	26 (100%)	0.238^a^
CDX2-positive	306 (92.2%)	22 (7.2%)	284 (92.8%)	
Histology	307	21 (6.8%)	286 (93.2%)	
Tumour grade 1 (Well differentiated)	24 (7.8%)	2 (8.3%)	22 (91.7%)	0.042^a^
Tumour grade 2 (Mod. differentiated)	255 (83.1%)	14 (5.5%)	241 (94.5%)	
Tumour grade 3 (Poorly differentiated)	28 (9.1%)	5 (17.9%)	23 (82.1%)	
Tumour sidedness	330	22 (6.7%)	308 (93.3%)	
Right sided	82 (24.8%)	4 (4.9%)	78 (95.1%)	0.622^a^
Left sided/Rectum	248 (75.2%)	18 (7.3%)	230 (92.7%)	
Tumour genetics				
*BRAF*	176	5 (2.8%)	171 (97.2%)	
Wild-type	161 (91.5%)	5 (3.1%)	156 (96.9%)	1.0^a^
p.V600E variant	15 (8.5%)	0 (0%)	15 (100%)	
*KRAS* or *NRAS*	332	22 (6.6%	310 (93.4%)	
Wild-type	172 (51.8%)	8 (4.7%)	164 (95.3%)	0.201^a^
Mutated	160 (48.2%)	14 (8.8%)	146 (91.2%)	
MMR	268	19 (7.1%)	249 (92.9%)	
MSS	259 (96.6%)	18 (6.9%)	241 (93.1%)	0.489^a^
High MSI	9 (3.4%)	1 (11.1%)	8 (88.9%)	
Sites of metastasis at diagnosis	332	22 (6.6%)	310 (93.4%)	
Liver	146 (44%)	11 (7.5%)	135 (92.5%)	0.77^a^
Peritoneum	72 (21.7%)	4 (5.6%)	68 (94.4%)	
Lung	56 (16.9%)	4 (7.2%)	52 (92.8%)	
Non regional lymph nodes	33 (9.9%)	3 (10.1%)	30 (90.9%)	
Bone	2 (0.6%)	0 (0.0%)	2 (100%)	
Other	23 (6.9%)	0 (0.0%)	23 (100%)	

Data presented as *n* (%)

a Chi-square test

MMR: mismatch repair; MSS: microsatellite stability; MSI: microsatellite instability

**Table 4.** CDX2 – Cox regression analysis for OS, both cohorts (*n* = 346 in multiple analysis).

Parameter	Univariate analyses		Multiple analyses	
	HR (95% CI)	*p* value	HR (95% CI)	*p* value
CDX2				
Positive	1 (reference)	0.010	1 (reference)	0.48
Negative	1.980 (1.178–3.327)		1.443 (0.525–3.967)	
*BRAF*				
Wild-type	1 (reference)	<0.001	1 (reference)	0.03
p.V600E	3.617 (1.807–7.242)		3.07 (1.125–8.377)	
Tumour sidedness				
Left/rectum	1 (reference)	0.011	1 (reference)	0.94
Right	1.588 (1.112–2.268)		0.974 (0.49–1.937)	

## References

[1] Aasebø K, Dragomir A, Sundström M (2020). CDX2: a prognostic marker in metastatic colorectal cancer defining a better BRAF mutated and a worse KRAS mutated subgroup. Front Oncol.

[2] Albasri AM, Elkablawy MA, Hussainy AS (2018). Impact of cyclooxygenase-2 over-expression on the prognosis of colorectal cancer patients an experience from western Saudi Arabia. Saudi Med J.

[3] Baba Y, Nosho K, Shima K (2009). Relationship of CDX2 loss with molecular features and prognosis in colorectal cancer. Clin Cancer Res.

[4] Bae JM, Lee TH, Cho NY (2015). Loss of CDX2 expression is associated with poor prognosis in colorectal cancer patients. World J Gastroenterol.

[5] Bruun J, Sveen A, Barros R (2018). Prognostic, predictive, and pharmacogenomic assessments of CDX2 refine stratification of colorectal cancer. Mol Oncol.

[6] Cervantes A, Adam R, Roselló S (2023). Metastatic colorectal cancer: ESMO clinical practice guideline for diagnosis, treatment and follow-up ☆. Ann Oncol.

[7] Chawengsaksophak K, De Graaff W, Rossant J (2004). CDX2 is essential for axial elongation in mouse development. Proceedings of the National Academy of Sciences.

[8] Coskun M, Troelsen JT, Nielsen OH (2011). The role of CDX2 in intestinal homeostasis and inflammation. Biochim Biophys Acta Mol Basis Dis.

[9] Cremolini C, Antoniotti C, Moretto R (2017). First-line therapy for mCRC-the influence of primary tumour location on the therapeutic algorithm. Nat Rev Clin Oncol.

[10] Dalerba P, Sahoo D, Paik S (2016). CDX2 as a prognostic biomarker in stage II and stage III colon cancer. N Engl J Med.

[11] Di Nicolantonio F, Vitiello PP, Marsoni S (2021). Precision oncology in metastatic colorectal cancer – from biology to medicine. Nat Rev Clin Oncol.

[12] Elzagheid A, Emaetig F, Alkikhia L (2013). High cyclooxygenase-2 expression is associated with advanced stages in colorectal cancer. Anticancer Res.

[13] Gao N, White P, Kaestner KH (2009). Establishment of intestinal identity and epithelial-mesenchymal signaling by CDX2. Dev Cell.

[14] Graule J, Uth K, Fischer E (2018). CDX2 in colorectal cancer is an independent prognostic factor and regulated by promoter methylation and histone deacetylation in tumors of the serrated pathway. Clin Epigenetics.

[15] Greenhough A, Smartt HJM, Moore AE (2009). The COX-2/PGE2 pathway: key roles in the hallmarks of cancer and adaptation to the tumour microenvironment. Carcinogenesis.

[16] Guo RJ, Eun RS, Lynch JP (2004). The role of CDX proteins in intestinal development and cancer. Cancer Biol Ther.

[17] Hansen TF, Kjær-Frifeldt S, Eriksen AC (2018). Prognostic impact of CDX2 in stage II colon cancer: results from two nationwide cohorts. Br J Cancer.

[18] Itzkowitz SH, Yio X (2004). Inflammation and cancer – IV. Colorectal cancer in inflammatory bowel disease: the role of inflammation. Am J Physiol Gastrointest Liver Physiol.

[19] James R, Erler T, Kazenwadel J (1994). Structure of the murine homeobox gene cdx-2. Expression in embryonic and adult intestinal epithelium. J Biol Chem.

[20] Kaimaktchiev V, Terracciano L, Tornillo L (2004). The homeobox intestinal differentiation factor CDX2 is selectively expressed in gastrointestinal adenocarcinomas. Mod Pathol.

[21] Kampf C, Olsson I, Ryberg U (2012). Production of tissue micro arrays, immunohisto chemistry staining and digitalization within the human protein atlas. J Vis Exp.

[22] Kim NK, Park JK, Shin E (2014). The combination of nuclear factor kappa B, cyclo-oxygenase-2 and vascular endothelial growth factor expression predicts poor prognosis in stage II and III colorectal cancer. Anticancer Res.

[23] Kim SH, Ahn BK, Paik SS (2018). Cyclooxygenase-2 expression is a predictive marker for late recurrence in colorectal cancer. Gastroenterol Res Pract.

[24] Kosumi K, Hamada T, Zhang S (2019). Prognostic association of PTGS2 (COX-2) over-expression according to BRAF mutation status in colorectal cancer: results from two prospective cohorts and CALGB 89803 (Alliance) trial. Eur J Cancer.

[25] Loupakis F, Biason P, Prete AA (2019). CK7 and consensus molecular subtypes as major prognosticators in V600E BRAF mutated metastatic colorectal cancer. Br J Cancer.

[26] Lugli A, Tzankov A, Zlobec I (2008). Differential diagnostic and functional role of the multi-marker phenotype CDX2/CK20/CK7 in colorectal cancer stratified by mismatch repair status. Mod Pathol.

[27] Macdonald PM, Struhl G (1986). A molecular gradient in early Drosophila embryos and its role in specifying the body pattern. Nature.

[28] Mahmoud AS, Umair A, Azzeghaiby SN (2014). Expression of cyclooxygenase-2 (COX-2) in colorectal adenocarcinoma: an immunohistochemical and histopathological study. Asian Pac J Cancer Prev.

[29] Mutoh H, Hayakawa H, Sakamoto H (2007). Homeobox protein CDX2 reduces COX-2 transcription by inactivating the DNA-binding capacity of nuclear factor-κB. J Gastroenterol.

[30] Negi RR, Rana SV, Gupta V (2019). Over-expression of cyclooxygenase-2 in colorectal cancer patients. Asian Pac J Cancer Prev.

[31] Neumann J, Heinemann V, Engel J (2018). The prognostic impact of CDX2 correlates with the underlying mismatch repair status and BRAF mutational status but not with distant metastasis in colorectal cancer. Virchows Archiv.

[32] Nolte S, Zlobec I, Lugli A (2017). Construction and analysis of tissue microarrays in the era of digital pathology: a pilot study targeting CDX1 and CDX2 in a colon cancer cohort of 612 patients. J Pathol Clin Res.

[33] Ogino S, Kirkner GJ, Nosho K (2008). Cyclooxygenase-2 expression is an independent predictor of poor prognosis in colon cancer. Clin Cancer Res.

[34] Olsen J, Eiholm S, Kirkeby LT (2016). CDX2 downregulation is associated with poor differentiation and MMR deficiency in colon cancer. Exp Mol Pathol.

[35] Patel JN, Fong MK, Jagosky M (2019). Colorectal cancer biomarkers in the era of personalized medicine. J Pers Med.

[36] Peng L, Zhou Y, Wang Y (2013). Prognostic significance of COX-2 immunohistochemical expression in colorectal cancer: a meta-analysis of the literature. PLoS One.

[37] Piawah S, Venook AP (2019). Targeted therapy for colorectal cancer metastases: a review of current methods of molecularly targeted therapy and the use of tumor biomarkers in the treatment of metastatic colorectal cancer. Cancer.

[38] Sakamoto N, Feng Y, Stolfi C (2017). BRAFV600E cooperates with CDX2 inactivation to promote serrated colorectal tumorigenesis. ELife.

[39] Sandberg TP, Sweere I, Pelt GW (2019). Prognostic value of low CDX2 expression in colorectal cancers with a high stromal content – a short report. Cell Oncol.

[40] Sanz-Garcia E, Argiles G, Elez E (2017). BRAF mutant colorectal cancer: prognosis, treatment, and new perspectives. Ann Oncol.

[41] Sartore-Bianchi A, Trusolino L, Martino C (2016). Dual-targeted therapy with trastuzumab and lapatinib in treatment-refractory, KRAS codon 12/13 wild-type, HER2-positive metastatic colorectal cancer (HERACLES): a proof-of-concept, multicentre, open-label, phase 2 trial. Lancet Oncol.

[42] Seligmann JF, Fisher D, Smith CG (2017). Investigating the poor outcomes of BRAF-mutant advanced colorectal cancer: analysis from 2530 patients in randomised clinical trials. Ann Oncol.

[43] Shigematsu Y, Inamura K, Yamamoto N (2018). Impact of CDX2 expression status on the survival of patients after curative resection for colorectal cancer liver metastasis 11 Medical and Health Sciences 1112 Oncology and Carcinogenesis. BMC Cancer.

[44] Silberg DG, Swain GP, Suh ER (2000). CDX1 and CDX2 expression during intestinal development. Gastroenterology.

[45] Singh J, Ng R, Dubashi B (2022). Pattern of expression of CDX2 in colorectal cancer and its role in prognosis. J Cancer Res Ther.

[46] Soares A, Rodrigues D, Cipriano E (2020). P-143 lack of expression of CDX2: prognostic biomarker in stage IV colorectal cancer. Ann Oncol.

[47] Soumaoro LT, Uetake H, Higuchi T (2004). Cyclooxygenase-2 expression: a significant prognostic indicator for patients with colorectal cancer. Clin Cancer Res.

[48] Tarazona N, Gimeno-Valiente F, Gambardella V (2020). Detection of postoperative plasma circulating tumour DNA and lack of CDX2 expression as markers of recurrence in patients with localised colon cancer. ESMO Open.

[49] Tejpar S, Stintzing S, Ciardiello F (2017). Prognostic and predictive relevance of primary tumor location in patients with ras wild-type metastatic colorectal cancer retrospective analyses of the CRYSTAL and FIRE-3 trials. JAMA Oncol.

[50] Tomasello G, Barni S, Turati L (2018). Association of CDX2 expression with survival in early colorectal cancer: a systematic review and meta-analysis. Clin Colorectal Cancer.

[51] Tomozawa S, Tsuno NH, Sunami E (2000). Cyclooxygenase-2 overexpression correlates with tumour recurrence, especially haematogenous metastasis, of colorectal cancer. Br J Cancer.

[52] Tran B, Kopetz S, Tie J (2011). Impact of BRAF mutation and microsatellite instability on the pattern of metastatic spread and prognosis in metastatic colorectal cancer. Cancer.

[53] Werling RW, Yaziji H, Bacchi CE (2003). CDX2, a highly sensitive and specific marker of adenocarcinomas of intestinal origin: an immunohistochemical survey of 476 primary and metastatic carcinomas. Am J Surg Pathol.

[54] Zhang BY, Jones JC, Briggler AM (2017a). Lack of caudal-type homeobox transcription factor 2 expression as a prognostic biomarker in metastatic colorectal cancer. Clin Colorectal Cancer.

[55] Zhang BY, Jones JC, Briggler AM (2017b). Lack of caudal-type homeobox transcription factor 2 expression as a prognostic biomarker in metastatic colorectal cancer. Clin Colorectal Cancer.

[56] Zhang H, Sun XF (2002). Overexpression of cyclooxygenase-2 correlates with advanced stages of colorectal cancer. Am J Gastroenterol.

[57] Zlobec I, Bihl MP, Schwarb H (2010). Clinicopathological and protein characterization of BRAF- and K-RAS-mutated colorectal cancer and implications for prognosis. Int J Cancer.

[58] Zlobec I, Terracciano L, Jass J R (2007). Value of staining intensity in the interpretation of immunohistochemistry for tumor markers in colorectal cancer. Virchows Archiv.

[59] Kunzmann AT, Murray LJ, Cardwell CR (2013). PTGS2 (cyclooxygenase-2) expression and survival among colorectal cancer patients: a systematic review. Cancer Epidemiol Biomarkers Prev.

